# Effectiveness of Early Direct Oral Anticoagulant Monotherapy within One Year of Coronary Stent Implantation in Patients with Atrial Fibrillation: A Nationwide Population-Based Study

**DOI:** 10.3390/jcm12237487

**Published:** 2023-12-04

**Authors:** Youmi Hwang, Soyoon Park, Soohyun Kim, Sung-Hwan Kim, Yong-Seog Oh, Kiyuk Chang, Young Choi

**Affiliations:** 1 Division of Cardiology, Department of Internal Medicine, St. Vincent’s Hospital, College of Medicine, The Catholic University of Korea, Suwon 16247, Republic of Korea; youmi0607@catholic.ac.kr; 2 Cardiovascular Research Institute for Intractable Disease, College of Medicine, The Catholic University of Korea, Seoul 06591, Republic of Korea; syoon007@hotmail.com (S.P.); withsoohyunk@gmail.com (S.K.); sunghwan@catholic.ac.kr (S.-H.K.); oys@catholic.ac.kr (Y.-S.O.); kiyuk@catholic.ac.kr (K.C.); 3 Division of Cardiology, Department of Internal Medicine, Seoul St. Mary Hospital, College of Medicine, The Catholic University of Korea, Seoul 06591, Republic of Korea

**Keywords:** direct acting oral anticoagulant, factor Xa inhibitor, percutaneous coronary intervention, atrial fibrillation, antiplatelet agent

## Abstract

We evaluated the effectiveness of early direct oral anticoagulant (DOAC) monotherapy within one year after percutaneous coronary intervention (PCI) in patients with atrial fibrillation (AF) using Korean National Health Insurance Service data. AF patients who underwent PCI were included and divided into the DOAC monotherapy group and the combination therapy group (DOAC with an antiplatelet agent) based on the medications used at 6 months after PCI. A major adverse cardiovascular event (MACE) was defined as a composite of cardiovascular death, acute myocardial infarction (AMI), stroke, or systemic thromboembolic event between 6 and 12 months after PCI. In the overall study population, the DOAC dose reduction rate was high in both the monotherapy group (70.8%) and the combination therapy group (79.1%). After propensity score matching, the MACE incidence was not significantly different between the two groups (hazard ratio [HR] 1.42 [0.90–2.24]). The numerical trend for higher MACE in the monotherapy group was mainly driven by the difference in stroke incidence (HR 1.84 [0.97–3.46]). All-cause death (HR 1.29 [0.61–2.74] or the incidence of major bleeding (HR 1.07 [0.49–2.35]) results were similar in the two groups. In conclusion, early DOAC monotherapy was not significantly associated with MACE risk between 6 and 12 months after PCI.

## 1. Introduction

Atrial fibrillation (AF) is associated with a risk of cardioembolic events and over 80% of patients with AF should be treated with oral anticoagulant [1]. Coronary artery disease frequently coexists with AF, and it has reported that approximately 20% of AF patients undergo percutaneous coronary intervention (PCI) for obstructive coronary diseases [2]. Patients with an implanted coronary stent require dual or single antiplatelet agent therapy to prevent stent thrombosis [3]. Therefore, when a patient with AF undergoes PCI, both antiplatelet agent and anticoagulants would be indicated; however, due to the potential for excessive bleeding risk, the appropriate drug regimens and duration for these patients remain to be further investigated [4,5,6,7,8]. The dual combination therapy composed of a direct oral anticoagulant (DOAC) plus a single antiplatelet agent is a standard antithrombotic regimen within one year after PCI in AF patients, but up to 10% of patients experience clinically relevant bleeding events in this period [9,10,11]. An antithrombotic regimen that employs only a single oral anticoagulant may be beneficial in such patients. However, due to ischemic concerns related to the discontinuation of an antiplatelet agent, clinical trials of the oral anticoagulant monotherapy have only been conducted in patients who are stable for more than one year after PCI [12,13]. In these patients, DOAC monotherapy has demonstrated significantly reduced bleeding risks with similar efficacy compared with the dual combination therapy. Meanwhile, to date, there is a lack of data regarding the effectiveness of DOAC monotherapy within one year of PCI.

With the development of a drug-eluting stent (DES), the risk of stent thrombosis has significantly decreased, and several studies regarding patients without AF have reported that the early application of single antiplatelet therapy within one year after PCI could be safe [14,15,16]. Current guidelines recommend DOAC monotherapy from one year after PCI in AF patients, but also suggest considering the earlier initiation of DOAC monotherapy through the discontinuation of the antiplatelet agent at 6 months in patients at low ischemic or high bleeding risks [17,18,19]. However, clinical evidence supporting the recommendation is as yet insufficient. This study aimed to evaluate the efficacy and safety of early DOAC monotherapy between 6 and 12 months after PCI using DES, compared with a conventional dual combination therapy in AF population.

## 2. Materials and Methods

### 2.1. Data Sources

We conducted a retrospective study using patient data from Korean National Health Insurance Service (KNHIS) claims from 2009 to 2020. The KNHIS is a single national insurer and includes comprehensive information with overall medical coverage. This service has approximately 50 million individuals enrolled from the Republic of Korea. The data include sociodemographic data, medical expenses, and diagnoses encoded by the International Classification of Disease, Tenth Revision of Clinical Modification. The data cover a wide range of inpatient and outpatient clinic services, medical costs, pharmacy claims, and mortality information and are provided anonymously. This study was conducted according to the Declaration of Helsinki and was approved by the Institutional Review Board of the Seoul St. Mary Hospital, The Catholic University of Korea (Seoul, Republic of Korea; Institutional Review Board No. KC20ZISI0928). Because this study was retrospectively conducted and used only anonymously coded data, informed consent was waived, which was approved by the Institutional Review Board.

### 2.2. Study Population and Group Definition

**Figure 1:**
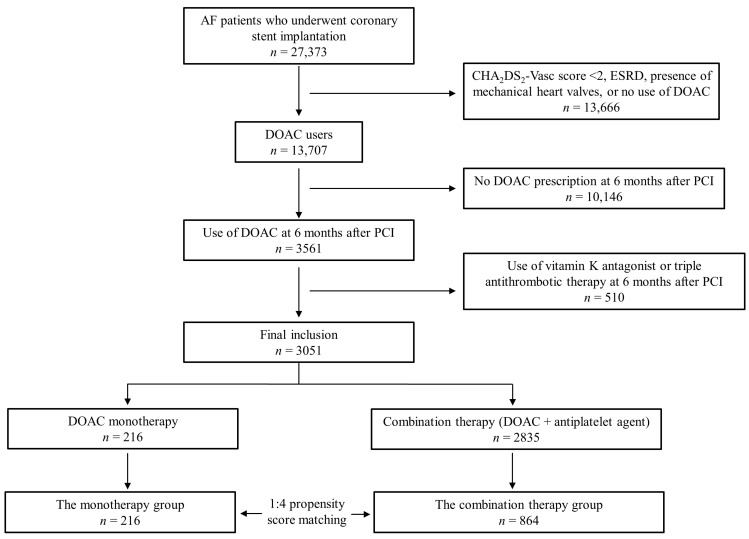
Flow chart for patient selection. AF = atrial fibrillation; ESRD = end-stage renal disease; DOAC = direct acting oral anticoagulant; PCI = percutaneous coronary intervention.

Based on all inpatient and outpatient claims, patients with AF who underwent percutaneous coronary stent implantation were searched. The inclusion criteria were (i) diagnosis of AF before index PCI; (ii) PCI with implantation of DES; and (iii) CHA_2_DS_2_-Vasc score ≥ 2 and use of DOAC at 6 months after PCI. Exclusion criteria were (i) age < 18 years; (ii) diagnosis of end-stage renal disease; (iii) use of a vitamin-K antagonist or dual antiplatelet agents at 6 months after PCI; and (iv) presence of mechanical valves. The detailed definitions of diagnoses and baseline covariates are provided in Supplemental Table S1. A total of 3051 patients were included in the final analyses. Patients were divided according to the prescribed antithrombotic medications at 6 months after PCI. The monotherapy group was defined as patients who were prescribed DOAC only, and the combination therapy group was defined as patients who were prescribed DOAC with a single antiplatelet agent. To balance the baseline covariates in the two groups, 1:4 propensity score (PS) matching was performed. The flowchart for patient selection and group classification is shown in Figure 1.

### 2.3. Study Outcomes

All outcomes of interest were assessed between 6 and 12 months after index PCI. The primary endpoint was a major adverse cardiovascular event (MACE), which was defined as a composite of cardiovascular death, myocardial infarction (MI), ischemic stroke, or systemic thromboembolic event. The secondary endpoints were all-cause deaths, major bleeding events, all critical anatomical site bleeding, and net adverse clinical events. Bleeding in a critical anatomical site was defined as newly diagnosed intracranial, intraocular, retroperitoneal, gastrointestinal, pericardial, intrathoracic, or intraarticular bleeding. A major bleeding event was defined as a critical anatomical site bleeding event that required hospitalization. Detailed definitions of the study outcomes are provided in Supplemental Table S2.

### 2.4. Statistical Analysis

Continuous variables are presented as means ± standard deviations and compared using Student’s *t*-tests. Categorical variables are presented as numbers and percentages and compared with the chi-square or Fisher’s exact test. Because the baseline characteristics of the two groups to be analyzed were expected to differ, we performed a PS matching analysis. PS was calculated for the covariates age, sex, chronic kidney disease, prior gastrointestinal bleeding, CHA_2_DS_2_-Vasc score, diagnosis at index PCI, DOAC dose reduction, use of renin–angiotensin system blockers, and use of beta blockers. The monotherapy and combination therapy groups were matched in a 1:4 ratio according to PS. An absolute difference (caliber) between the PS of 0.001 was applied, and the closest option was used to optimize the model. The cumulative incidence of MACE and the secondary endpoints were analyzed using Kaplan–Meier survival curves and compared using the log-rank test. Cox regression analyses were performed to assess the hazard ratios for each clinical outcome in the two groups after PS matching. As a sensitivity analysis, per-protocol analysis was conducted in the PS-matched population by censoring patients in outcome analyses when the index treatment drug was discontinued during the study period. Subgroup analyses were performed in the PS-matched population to assess the impact of early monotherapy on the risk of MACE in subgroups defined according to age, sex, diabetes mellitus, diagnosis at PCI, prior history of major bleeding, and DOAC dose reduction. All analyses were two-tailed, and a *p*-value < 0.05 was considered to indicate statistical significance. Statistical analyses were performed using SAS software V9.4 (SAS Institute, Cary, NC, USA).

## 3. Results

### 3.1. Baseline Characteristics

Baseline characteristics in the two groups before PS matching are presented in Table 1 (monotherapy group; *n* = 216, combination therapy group; *n* = 2835). The mean age was 74.5 (±8.5) years and 2011 (65.9%) were male, without a significant difference between the two groups. Among the comorbidities, the prevalence of chronic kidney disease and prior stroke was significantly higher in the monotherapy group, and previous MI was more prevalent in the combination therapy group. The mean CHA_2_DS_2_-Vasc score was higher in the monotherapy group than in the combination therapy group (6.1 ± 1.6 vs. 5.9 ± 1.6, respectively; *p* = 0.036). Prior history of intracranial hemorrhage or gastrointestinal bleeding was not significantly different between the two groups. The proportion of patients with acute MI at index PCI was 15.2% in the monotherapy group and 22.5% in the combination therapy group (*p* = 0.014). DOAC dose reduction was more frequent in the combination therapy group (79.1%) compared to the monotherapy group (70.8%) (*p* = 0.004).

After 1:4 PS matching, there were 216 patients in the monotherapy group and 864 in the combination therapy group. All baseline variables, including demographic characteristics, comorbidities, and concomitant medications, were well-balanced between the two groups after PS matching (Table 2). The mean CHA_2_DS_2_-Vasc score was 6.1 ± 1.6 in both groups. For the combination therapy group, the prescribed antiplatelet agent was mostly a P2Y12 inhibitor (87.2%).

### 3.2. Clinical Outcomes

**Figure 2:**
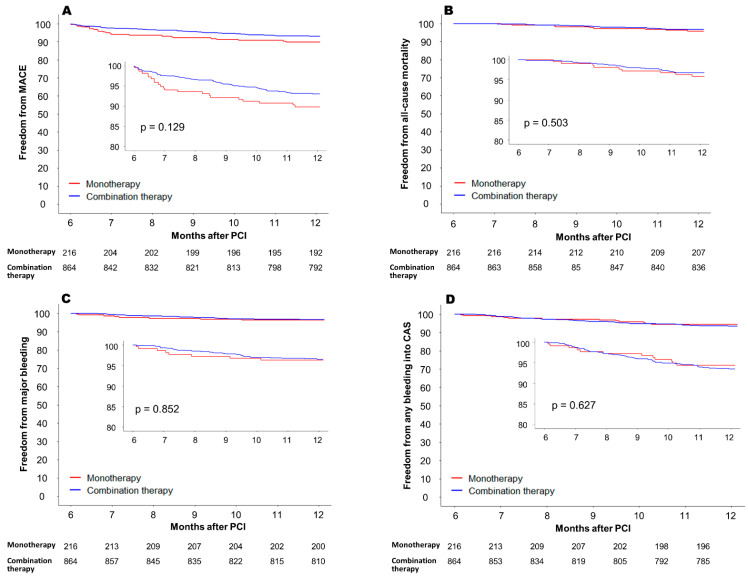
Clinical outcomes between 6 and 12 months after PCI in the propensity score-matched cohort. (**A**) Freedom from MACE, (**B**) freedom from all-cause mortality, (**C**) freedom from major bleeding (bleeding event requiring hospitalization, and (**D**) freedom from any bleeding into CAS. MACE = major adverse cardiovascular event; PCI = percutaneous coronary intervention; CAS = critical anatomical site.

Study outcomes were compared across the PS-matched groups. The incidence of MACE between 6 and 12 months after PCI was not significantly different between the two groups (Table 3). However, there was a trend towards higher MACE incidence in the monotherapy group (10.6% vs. 6.9% in the monotherapy group and combination therapy group, respectively; hazard ratio [HR] = 1.42, 95% confidence interval [CI] 0.90–2.24, *p* = 0.129) (Figure 2A). Among the individual components of MACE, the incidence of ischemic stroke mostly differed between the two groups, but the difference did not reach statistical significance (6.5% vs. 3.6%, respectively; HR = 1.84, 95% CI 0.97–3.46, *p* = 0.058). The incidence of cardiovascular death and MI was comparable in the two groups. In the secondary endpoint analyses, all-cause mortality was not significantly different between the two groups (4.1% vs. 3.2%, respectively, HR = 1.29, 95% CI 0.61–2.74, *p* = 0.503) (Figure 2B). The incidence of a major bleeding event was similar in the two groups (3.7% vs. 3.5%, respectively, HR 1.07, 95% CI 0.49–2.35, *p* = 0.852) (Figure 2C). Any critical anatomical site bleeding occurred in 12 (5.6%) in the monotherapy group and 56 (6.5%) in the combination therapy group (HR 0.86, 95% CI 0.46–1.60, *p* = 0.627) (Figure 2D).

### 3.3. Subgroup Analysis and Per-Protocol Treatment Analysis

**Figure 3:**
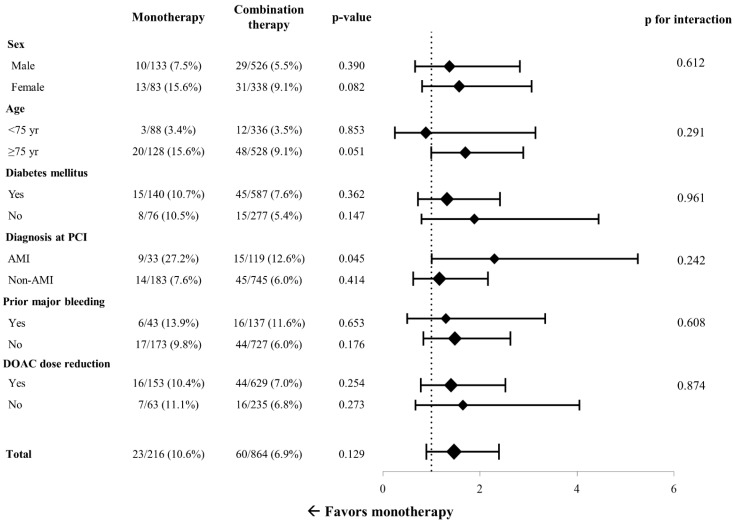
Subgroup analysis for risk of major adverse cardiac events. PCI = percutaneous coronary intervention; DOAC = direct oral anticoagulant.

**Figure 4:**
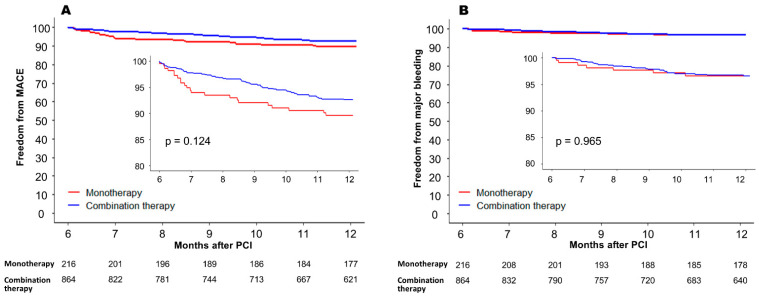
Per-protocol treatment comparison between 6 and 12 months after PCI in the propensity-score-matched cohort. (**A**) MACE-free survival, and (**B**) freedom from major bleeding event. MACE = major adverse cardiovascular event; PCI = percutaneous coronary intervention.

In the subgroup analysis, significant interaction was not demonstrated between the effect of DOAC monotherapy on MACE risk and all prespecified subgroups classified by age, sex, diabetes mellitus, prior major bleeding, diagnosis at index PCI, and DOAC dose reduction (Figure 3). However, when separately assessed according to the diagnosis at the time of index PCI, the MACE rate was higher with DOAC monotherapy with a marginal statistical significance in patients who presented with acute MI (HR = 2.32, 95% CI 1.01–5.32, *p* = 0.045), while it was similar between the two groups in those who presented with non-acute MI (HR = 1.28, 95% CI 0.70–2.33, *p* = 0.414). The per-protocol treatment comparison was additionally conducted as a sensitivity analysis. The incidence of MACE between 6 and 12 months after PCI was not significantly different between the monotherapy group and the combination therapy group (10.2% vs. 6.8%, HR = 1.46, 95% CI 0.89–2.39, *p* = 0.125) (Figure 4A). Also, all-cause mortality (4.1% vs. 3.2%, respectively, HR = 1.23, 95% CI 0.57–2.59, *p* = 0.599) and the incidence of major bleeding events (3.2% vs. 3.1%, respectively, HR 1.01, 95% CI 0.44–2.33, *p* = 0.965) were not significantly different in the two groups (Figure 4B).

## 4. Discussion

We assessed the efficacy and the safety of early DOAC monotherapy between 6 and 12 months after PCI using a DES in a nationwide population-based dataset. The MACE rate was not significantly different between the early DOAC monotherapy group and the combination therapy group. There was a numerical trend towards a higher MACE rate with the monotherapy, which was predominantly driven by stroke events. According to the diagnosis at the time of index PCI, the MACE incidence was similar between the monotherapy and combination therapy groups in non-acute MI patients, while the MACE incidence was increased with the monotherapy in acute MI patients, although the statistical significance was minimal. The rates of bleeding events that required hospitalization and critical anatomical site bleeding were similar between the monotherapy and the combination therapy groups.

### 4.1. Ischemic Event Risk with Early DOAC Monotherapy

Evidence to date suggests a shorter duration of combination therapy with a preference for DOAC over vitamin K antagonists for anticoagulants for patients undergoing PCI, due to bleeding risks [17,18,19,20,21]. The introduction of better-profiled DES contributed to lower risks of restenosis and stent thrombosis [22] and a trend towards a shorter duration of dual antiplatelet therapy after PCI in patients without AF [23,24]. In patients with AF, combination therapy is recommended for up to one year post-PCI, followed by a transition to DOAC monotherapy [17,18]. However, when earlier DOAC monotherapy within one year is considered, there is a lack of data regarding the optimal timing for the switching. To our knowledge, our study is the first to show real-world data of early DOAC monotherapy from 6 months after PCI in an AF population, and showed that this strategy would be effective in terms of MACE prevention within one year. Most patients included in our study had received PCI for non-acute MI, and overall ischemic event was comparable in the two groups. However, in the small number of patients with acute MI who received early monotherapy, the MACE incidence was unexpectedly high. The heterogeneity in the effect of DOAC monotherapy in MI versus non-MI patients has not been previously demonstrated in studies concerning the AF-PCI population [12,13,25]. In the AFIRE study, which enrolled AF patients who were stable for more than one year after PCI, the risk of ischemic events was even lower in the DOAC monotherapy group compared to the combination therapy group, without heterogeneity in all subgroups [12]. A previous study conducted in the Korean AF-PCI population also showed that DOAC monotherapy did not increased the risk of MACE one year after PCI, regardless of the presence of prior MI [13]. Currently, it is recommended to maintain dual antiplatelet therapy for one year after acute coronary syndrome (ACS) [19]. Han et al. reported that 6-month dual antiplatelet therapy was associated with an increased risk of MI, compared to the 12-month dual antiplatelet therapy in patients with ACS [26]. Meanwhile, since the majority of MACE in our study were attributable to strokes, it is unlikely that the ischemic event risk associated with coronary lesions has increased due to monotherapy in patients with acute MI. The efficacy of early DOAC monotherapy in patients with a high ischemic risk warrants further validation in a cohort including a larger population of patients with ACS.

### 4.2. Effect of Dose Reduction of DOAC

It should also be taken into consideration that the rate of off-label dose reduction of DOAC in the monotherapy group of our study was approximately 70%. The exact reason for the high rate of DOAC dose reduction is not clear; a plausible explanation is that physicians would not have been willing to administer DOAC at standard doses when discontinuing antiplatelet agents early in patients believed to be at high risk of bleeding. Although DOAC dose reduction is not recommended during combination with an antiplatelet agent [17,18], the rate of a dose reduction of DOAC was also high (79%) in the combination therapy group. The off-label underdosing of DOAC has been shown to increase the risk of thromboembolism, and co-prescription with an antiplatelet agent is generally one of the common reasons [27]. This may also have contributed to the high DOAC dose reduction rate in the monotherapy group, as the patients would have previously received combination therapy immediately after PCI, and only then discontinued the antiplatelet agent. We found that ischemic stroke was the most frequent ischemic complication in this period, and the results indicate the greater need for appropriate dosing of oral anticoagulants for this population.

### 4.3. Bleeding Risks with the Early DOAC Monotherapy

The incidence of major bleeding was not significantly reduced in the DOAC monotherapy group in our study. We defined major bleeding as bleeding in a critical anatomical site that requires hospitalization, according to the widely adopted bleeding definition of the International Society on Thrombosis and Hemostasis (ISTH) [27]. However, the definition of bleeding in this study could not be completely concordant with the ISTH criteria due to limitations in the cohort data. In order to assess major bleeding events that are either life-threatening or clinically relevant, necessitating intensive medical care, we specifically included only those requiring hospitalization. This definition may have underestimated the incidence of major bleeding. However, the observed incidence of major bleeding (3.1% in 6 months) in the combination therapy group is consistent with previous studies reporting major bleeding event rates of 3–6% per year [9,11,12]. In previous studies, DOAC monotherapy has been shown to reduce major bleeding events in patients with stable coronary artery disease [12,13]. The discrepancy in our study might be attributable to the small number of included patients and a short follow-up duration, as well as the bias owing to the difference in baseline bleeding risks in the two groups. Meanwhile, we found a trend towards a lower incidence of any bleeding events (either requiring hospitalization or not) in a critical anatomical area in the monotherapy group. Considering that the risk of bleeding events remains significant even during a short follow-up period of 6 months, the effect of earlier monotherapy switching in reducing clinically relevant bleeding should be further evaluated.

### 4.4. Clinical Implication

Although early DOAC monotherapy did not significantly increase the MACE incidence within one year post-PCI in our study, this strategy should be applied cautiously in patients at high-ischemic risk. Despite the technical evolution of PCI, there are still concerns about stent thrombosis or recurrent ischemic cardiovascular events with a shorter duration or fewer numbers of antiplatelet agents [28,29]. Patient characteristics, such as comorbidities associated with a higher risk of thrombotic/ischemic events, PCI due to ACS, residual untreated coronary lesions, and the complexity of PCI lesions, should be considered when reducing the intensiveness of antithrombotic therapy [30]. In our study, DOAC monotherapy was not associated with increased acute coronary events or cardiovascular death, without a significant interaction with the acute MI subgroup. Therefore, not only the presence of ACS but also a range of other factors should be considered comprehensively in defining high-ischemic risk groups concerning early DOAC monotherapy. In patients with low ischemic risk, our study data indicate that early DOAC monotherapy does not increase ischemic events within one year. However, this study is based on real-world data, and the monotherapy group is more likely to represent high-bleeding risk groups rather than a general AF-PCI population. An ongoing, randomized trial (OPTIMA-AF) would provide valuable information regarding the efficacy and safety of early DOAC monotherapy within one year [31].

### 4.5. Limitations

First, this study is retrospective and the number of included patients may not be sufficient to statistically validate the difference in clinical outcomes. Nevertheless, this study was conducted with the largest number of patients that could be obtained through national health insurance data to date. Second, this was a retrospective study, and the prevalence of chronic kidney disease, stroke, prior MI, and CHA_2_DS_2_-Vasc score were different in the two groups at baseline. Although we performed PS matching, there may have been overlooked or unmeasurable confounders despite adjusting for various covariates. Additionally, the study outcomes were observed from 6 months post-PCI; however, the onset of exposure to the treatment for each group may precede this period. This design entails the potential for immortal time bias. Third, the frequencies of DOAC dose reduction were unexpectedly high. This might be due to the physician’s preference for low-dose DOAC for a combination with antiplatelet agent and for high-bleeding risk patients. Consequently, the proportion of stroke events was also high, which may be attributable to the underdosing of DOACs. While this reflects real-world practice, it would be a significant limitation of this study. Fourth, the rate of unplanned revascularization owing to non-acute MI was not analyzed. Although it would be of interest to clinicians, this outcome appeared unsuitable for analysis in this cohort because it was unable to distinguish between planned and unplanned revascularization in this retrospective data, and the revascularization plan itself was likely to influence the drug regimen. Fifth, the definition of bleeding in this study was not consistent with other standard definitions such as those of TIMI or ISTH, and thus the effect of antithrombotic therapy on bleeding outcome might have been underestimated. Finally, we could not assess the complexity of index PCI/target lesions or the non-treated coronary artery disease status, which is crucial to determine individual ischemic risks.

## 5. Conclusions

In this nationwide, population-based cohort study, the incidences of MACE and the major bleeding events requiring hospitalization between 6 and 12 months after PCI in AF patients were not significantly different between the early DOAC monotherapy group and the combination therapy group. There was a nonsignificant trend towards a higher incidence of MACE in the monotherapy group, which was predominantly driven by the difference in stroke events. The rate of DOAC underdosing was notably high in this real-world data, suggesting that the appropriate dosing of DOACs is critical in this population, especially when implementing DOAC monotherapy. Whether or not early DOAC monotherapy confers net clinical benefits in AF patients who underwent PCI should be confirmed through a future, well-designed prospective study.

## Figures and Tables

**Table 1:** Baseline characteristics in the two groups before propensity score matching.

	Monotherapy (*n* = 216)	Combination Therapy (*n* = 2835)	*p*-Value
Age, years	75.5 ± 8.7	74.4 ± 8.5	0.090
Male, *n* (%)	133 (61.5%)	1878 (66.2%)	0.162
Comorbidities, *n* (%)			
Hypertension	185 (85.6%)	2551 (90.0%)	0.057
Diabetes mellitus	140 (64.8%)	1883 (66.4%)	0.630
Heart failure	167 (77.3%)	2056 (72.5%)	0.126
Chronic kidney disease	75 (34.7%)	780 (27.5%)	0.023
Prior history of stroke	100 (46.3%)	1102 (38.8%)	0.031
Prior history of MI	66 (30.5%)	1095 (38.6%)	0.018
Liver cirrhosis	4 (1.8%)	91 (3.2%)	0.268
Prior history of ICH	9 (4.1%)	90 (3.1%)	0.427
Prior history of GI bleeding	63 (29.1%)	797 (28.1%)	0.740
Prior CABG	0	7 (0.25%)	0.467
CHA_2_DS_2_-Vasc score	6.1 ± 1.6	5.9 ± 1.6	0.036
Diagnosis at index PCI			0.014
Non-AMI, *n* (%)	183 (84.7%)	2198 (77.5%)	
AMI, *n* (%)	33 (15.2%)	637 (22.5%)	
DOAC dose reduction, *n* (%)	153 (70.8%)	2241 (79.1%)	0.004
Antiplatelet agent type			
Aspirin		361 (12.7%)	
P2Y12 inhibitor		2474 (87.3%)	
Other medications, *n* (%)			
ACEi/ARB	172 (79.6%)	2148 (75.7%)	0.199
Statin	179 (82.8%)	2390 (84.3%)	0.577
Beta blocker	179 (82.8%)	2223 (78.4%)	0.122

Categorical variables are presented as numbers (percentages) and continuous variables are presented as mean ± standard deviation. *p* < 0.05 indicates statistical significance. MI = myocardial infarction; ICH = intracranial bleeding; GI = gastrointestinal; CABG = coronary artery bypass graft; PCI = percutaneous coronary intervention; AMI = acute myocardial infarction; DOAC = direct oral anticoagulant; ACEi = angiotensin converting enzyme inhibitor; ARB = angiotensin receptor blocker.

**Table 2:** Baseline characteristics in the two groups after propensity score matching.

	Monotherapy (*n* = 216)	Combination Therapy (*n* = 864)	*p*-Value	SMD
Age, years	75.5 ± 8.7	75.6 ± 8.3	0.861	0.013
Male, *n* (%)	133 (61.5%)	526 (60.9%)	0.851	0.014
Comorbidities, *n* (%)				
Hypertension	185 (85.6%)	763 (88.3%)	0.341	0.192
Diabetes mellitus	140 (64.8%)	587 (67.9%)	0.381	0.066
Heart failure	167 (77.3%)	657 (76.0%)	0.693	0.029
Chronic kidney disease	75 (34.7%)	281 (32.5%)	0.538	0.046
Prior history of stroke	100 (46.3%)	362 (41.9%)	0.242	0.088
Prior history of MI	66 (30.5%)	275 (31.8%)	0.718	0.027
Liver cirrhosis	4 (1.8%)	22 (2.5%)	0.551	0.045
Prior history of ICH	9 (4.1%)	26 (3.0%)	0.390	0.065
Prior history of GI bleeding	63 (29.1%)	235 (27.2%)	0.562	0.043
Prior CABG	0	0	NS	
CHA_2_DS_2_-Vasc score	6.1 ± 1.6	6.1 ± 1.6	0.918	0.007
Diagnosis at index PCI			0.569	0.043
Non-AMI, *n* (%)	183 (84.7%)	745 (86.2%)		
AMI, *n* (%)	33 (15.2%)	119 (13.7%)		
DOAC dose reduction, *n* (%)	153 (70.8%)	629 (72.8%)	0.562	0.043
Antiplatelet agent type				
Aspirin		111 (12.8%)		
P2Y12 inhibitor		753 (87.2%)		
Other medications, *n* (%)				
ACEi/ARB	172 (79.6%)	689 (79.7%)	0.969	0.002
Statin	179 (82.8%)	724 (83.8%)	0.742	0.024
Beta blocker	179 (82.8%)	725 (83.9%)	0.710	0.028

Categorical variables are presented as numbers (percentages) and continuous variables are presented as mean ± standard deviation. *p* < 0.05 indicates statistical significance. SMD = standardized mean difference; MI = myocardial infarction; ICH = intracranial bleeding; GI = gastrointestinal; CABG = coronary artery bypass graft; PCI = percutaneous coronary intervention; AMI = acute myocardial infarction; DOAC = direct oral anticoagulant; ACEi = angiotensin converting enzyme inhibitor; ARB = angiotensin receptor blocker.

**Table 3:** The primary and secondary endpoints in the propensity-score-matched population.

	Monotherapy (*n* = 216)	Combination Therapy (*n* = 864)	Hazard Ratio	95% CI	*p*-Value
MACE	23 (10.6%)	60 (6.9%)	1.42	0.90–2.24	0.129
Cardiovascular death	4 (1.8%)	15 (1.7%)	1.07	0.34–3.09	0.900
Myocardial infarction	5 (2.3%)	19 (2.2%)	1.06	0.39–2.82	0.913
Ischemic stroke	14 (6.5%)	31 (3.6%)	1.84	0.97–3.46	0.058
Systemic thromboembolic event	2 (0.9%)	6 (0.7%)	1.34	0.27–6.66	0.716
All-cause death	9 (4.1%)	28 (3.2%)	1.29	0.61–2.74	0.503
Major bleeding *	8 (3.7%)	30 (3.5%)	1.07	0.49–2.35	0.852
Intracranial bleeding	0	0			
Gastrointestinal bleeding	6 (2.8%)	21 (2.4%)	1.15	0.46–2.85	0.760
Other critical area bleeding	2 (0.9%)	10 (1.1%)	0.80	0.17–3.66	0.778
Any critical anatomical site bleeding	12 (5.6%)	56 (6.5%)	0.86	0.46–1.60	0.627

* Defined as critical area or organ bleeding that required hospitalization. MACE = major adverse cardiovascular event; CI = confidence interval.

## Data Availability

The datasets analyzed in this study are not publicly available.

## Supplementary Materials

The following supporting information can be downloaded at: https://www.mdpi.com/article/10.3390/jcm12237487/s1, Table S1: Definitions of baseline covariates. Table S2. Definitions of study outcomes.
