# Supplementary material for: Effectiveness of Early Direct Oral Anticoagulant Monotherapy within One Year of Coronary Stent Implantation in Patients with Atrial Fibrillation: A Nationwide Population-Based Study
Source: J Clin Med. 2023 Dec 4;12(23):7487. doi: 10.3390/jcm12237487 (PMC10706918; doi:10.3390/jcm12237487)
Supplement: Supplementary file 1 [file jcm-12-07487-s001.zip › jcm-2692137-supplementary.pdf]

Supplemental Table S1. Definitions of baseline covariates

Diagnosis	ICD-10-CM code or prescription code	Diagnostic definition
**Atrial fibrillation**	I48	Admission≥1 or outpatient department≥2
**Hypertension**	I10-I13, I15	Admission≥1 or outpatient department≥2
**Diabetes mellitus**	E10-E14	Admission≥1 or outpatient department≥2
**Heart failure**	I11.0, I13.0, I13.2, I42, I50	Admission≥1 or outpatient department≥2
**Chronic kidney disease**	I12, I13, N00-05, N07, N11, N14, N17-19, Z49, Q61	Admission or outpatient department≥1
**End-stage renal disease**	N185, Z49	Admission or outpatient department≥1
**Liver cirrhosis**	K74, K702, K703, K717, K761	Admission or outpatient department≥1
**Myocardial infarction**	I21, I22, I23	Admission≥1 or outpatient department≥2
**Ischemic stroke**	I63, I64	Admission≥1 or outpatient department≥2
**Intracranial hemorrhage**	I60-62	Admission≥1 or outpatient department≥2
**GI bleeding**	I85.0, K22.1, K22.8, K25.0, K25.2, K25.4, K25.6, K26.0, K26.2, K26.4, K26.6, K27.0, K27.2, K27.4, K27.6, K28.0, K28.2, K28.4, K28.6, K29.0, K31.8, K55.2, K57.0, K57.1, K57.2, K57.3, K57.4, K57.5, K57.8, K57.9, K62.5, K66.1, K92.0, K92.1, K92.2	Admission≥1 or outpatient department≥2
**PCI**	M6561, M6562, M6563, M6564, M6565, M6566	Operation codes
**CABG**	O1640, O1641, O1645, O1646, O1647, O1648, O1649	Operation codes
**DOAC dose**		
**Low dose**		Dabigatran 110mg, rivaroxaban

		10mg/15mg, apixaban 2.5mg, or edoxaban 15mg/30mg
**Standard dose**		Dabigatran 150mg, rivaroxaban 20mg, apixaban 5mg, or edoxaban 60mg
**Drug-eluting stent**	J5083001, J5083002, J5083011, J5083029, J5083040, J5083046, J5083070, J5083101, J5083137, J5083140, J5083173, J5083201, J5083213, J5083237, J5083240, J5083273, J5083301, J5083401, J5083501, J5231221, J5231294, J8083033, J8083089, J8083129, J8083340, J8083440, J8083473, J5231306, J5231045, J5231065, J5083006, J5083013, J5083042, J5083073, J5083113, J8083073, J8083373	Material codes at the time of PCI

GI = gastrointestinal; PCI = percutaneous coronary intervention; CABG = coronary artery bypass graft; DOAC = direct oral anticoagulant.

Supplemental Table S2. Definitions of study outcomes

Outcomes	ICD-10-CM code	Diagnostic definition
**MACE**		A composite event of cardiovascular death, myocardial infarction, ischemic stroke or systemic thromboembolism
**Cardiovascular death***	I00.X-I99.X or R96, R98, R99	Recorded as a major cause of death
**Myocardial infarction**	I21, I22, I23	Major diagnosis for admission during the study period
**Ischemic stroke**	I63, I64	Major diagnosis for admission during the study period
**Systemic thromboembolism**	I74, I26, I80.2	Major diagnosis for admission during the study period
**Major bleeding**		Major diagnosis of ICH, GI bleeding or other critical area bleeding for admission during the study period
**ICH**	I60-62	Major diagnosis for admission during the study period
**GI bleeding**	I85.0, K22.1, K22.8, K25.0, K25.2, K25.4, K25.6, K26.0, K26.2, K26.4, K26.6, K27.0, K27.2, K27.4, K27.6, K28.0, K28.2, K28.4, K28.6, K29.0, K31.8,	Major diagnosis for admission during the study period

	K55.2, K57.0, K57.1, K57.2, K57.3, K57.4, K57.5, K57.8, K57.9, K62.5, K66.1, K92.0, K92.1, K92.2	
**Other critical area bleeding**	D62, H05.2, H35.6, H43.1, J94.2, M25.0, I312, I319	Major diagnosis for admission during the study period
**Any critical anatomical site bleeding**		New diagnosis of ICH, GI bleeding or other critical area bleeding for either admission or outpatient visit, during the study period

*Causes of deaths were identified by the data provided from the Korean national statistical office.

MACE = major adverse cardiovascular event; ICH = intracranial hemorrhage; GI = gastrointestinal.
